# Assessment of abdominal lesions in pigs with wounded umbilical outpouchings transported to slaughter

**DOI:** 10.1186/s40813-024-00371-z

**Published:** 2024-05-17

**Authors:** Benjamin Meyer Jørgensen, Tina Birk Jensen, Cecilie Brandt Becker, Christoffer Kirkelund Flyger, Maja Vive Christensen, Andreas Birch, Henrik Elvang Jensen

**Affiliations:** 1 Department of Veterinary and Animal Sciences, Section for Pathobiological Sciences, University of Copenhagen, Grønnegårdsvej 15, Frederiksberg C, 1870 Denmark; 2 SEGES Innovation, Agro Food Park 15, Aarhus, 8200 Denmark; 3 Ø-Vet A/S, Køberupvej 33, Naestved, 4700 Denmark

**Keywords:** Animal welfare, Umbilical outpouchings, Hernia, Enterocystoma, Pig

## Abstract

**Background:**

Umbilical outpouchings (UOs) in pigs are of welfare and production concern in Danish pig herds. One of the challenges is the transportation of these animals due to the size of the UOs and ulcerations on them. With certain precautions and an approval from a veterinarian, pigs with UOs may be transported, however, UOs are associated with several intra-abdominal lesions, e.g. peritonitis and incarceration, which may worsen during the process of transportation. The prevalence and characterization of intra-abdominal lesions associated with UOs following transportation has not been investigated. Therefore, the objective of the present study was to evaluate lesions associated with the intestines and peritoneum in slaughter pigs with wounded UOs following transportation to an abattoir.

**Results:**

A cross-sectional study involving three conventional Danish pig herds was conducted comprising 96 slaughter pigs with wounded UOs transported to an abattoir. Following slaughter the UOs with accompanying intestines were examined pathologically. Three distinct morphological categories were present: hernia, enterocystoma and herniating enterocystoma. Intra-abdominal lesions were present in 72% of the animals, representing 65% (44/68) of the hernias, 77% (10/13) of the enterocystomas, and 100% (15/15) of the herniating enterocystomas. Several different lesions were found like bleedings, acute/chronic peritonitis and hypertrophy of the intestinal muscular layers.

**Conclusions:**

The prevalence of intra-abdominal lesions in pigs transported with a wounded UO was found to be high independent of the underlying condition, and it is clear that these pigs possess a vulnerable group of animals, especially during physical stressful situations like transportation. More information is needed regarding the prevalence of intra-abdominal lesions in UO pigs without wounds on the UO. The results provide valuable knowledge, that can be used when examining and evaluating UO pigs before transportation.

## Background

In Danish conventional pig herds the prevalence of umbilical outpouchings (UOs) is 4.2% [1], and with about 32 million piglets produced annually in Denmark, this equates to approximately 1.4 million pigs with an UO [2, 3]. It has been estimated that 1.3% of pigs have an UO at the time of slaughter [4]. Various factors may be responsible for this decrease in prevalence. However, the main cause is likely unfitness for transportation, death or euthanasia, due to complications relating to the UOs. In addition, both the size and the presence of ulcerations on the surface of the UOs, play an important role, since both factors affect the eligibility for transport according to the Danish legislation [3]. According to the Danish Veterinary Health Council, pigs with wounds on the UOs are not fit for transport in Denmark regardless of the severity of the wound.

In Denmark, pigs are by the Animal Welfare Act § 2 [5] protected against pain, suffering, anxiety, lasting harm, and significant inconvenience. Moreover, in the European and Danish transport order [6], it is directed that slightly injured or sick animals can be transported when certain precautions are taken (extra space and soft bedding) and the animal has been deemed eligible for transportation by a veterinary clinician. However, it may be difficult to approve pigs with UOs to be fit for transportation due to the various underlying causes of UOs in pigs. Even though a pig with an UO seems eligible for transportation from the clinical examination, the impact of transportation may be stressful and can lead to worsening health and death of the animal, as several pathological complications may derive from having an UO, e.g. peritonitis, intestinal incarceration, and hemorrhages [7, 8]. Therefore, it is most likely that such intra-abdominal lesions may be exacerbated by the physical impact before, during, and after transportation to the slaughterhouse, potentially resulting in increased suffering and distress. However, the prevalence of UO-associated pathology following transportation has not been investigated. Therefore, the objective of the present study was to evaluate lesions associated with the intestines and peritoneum in slaughter pigs with wounded UOs following transportation to an abattoir.

## Results

**Fig. 1:**
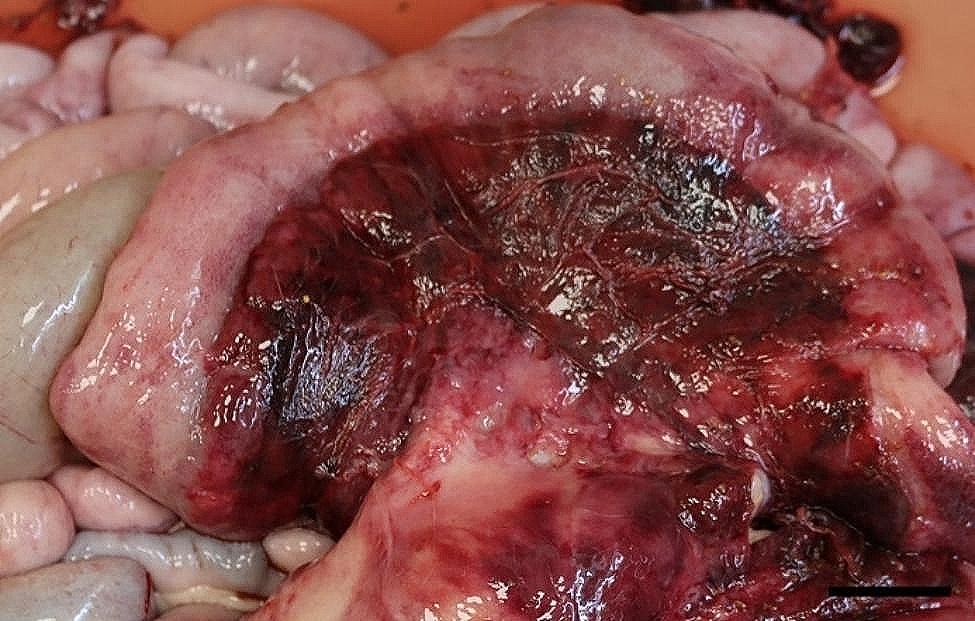
Widespread hemorrhages in the jejunal mesentery of a slaughter pig with an umbilical hernia. Bar = 4 cm

**Fig. 2:**
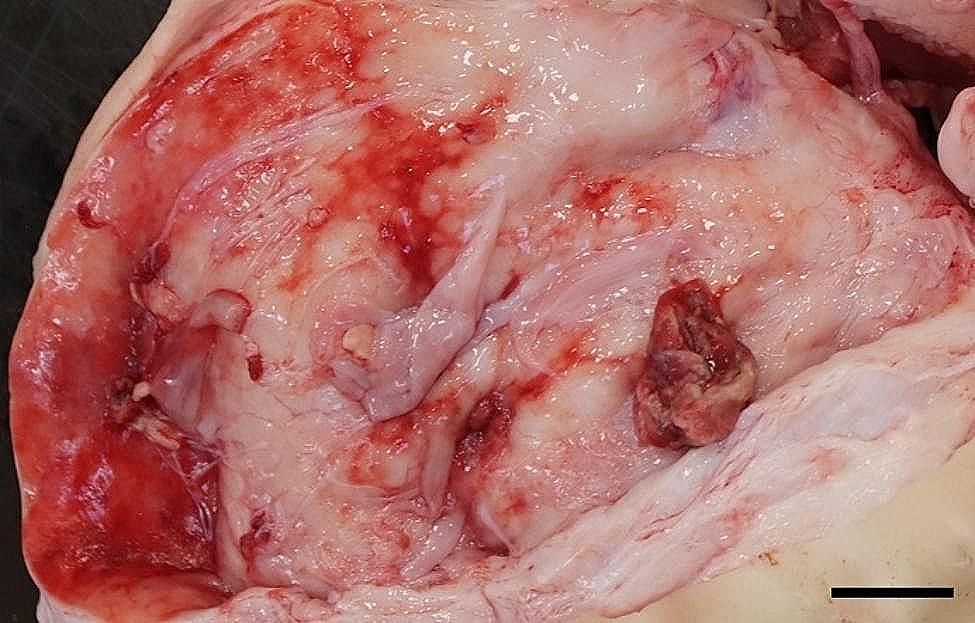
Widespread hemorrhages in the peritoneal lining of an umbilical hernia of a slaughter pig. Bar = 4 cm

**Fig. 3:**
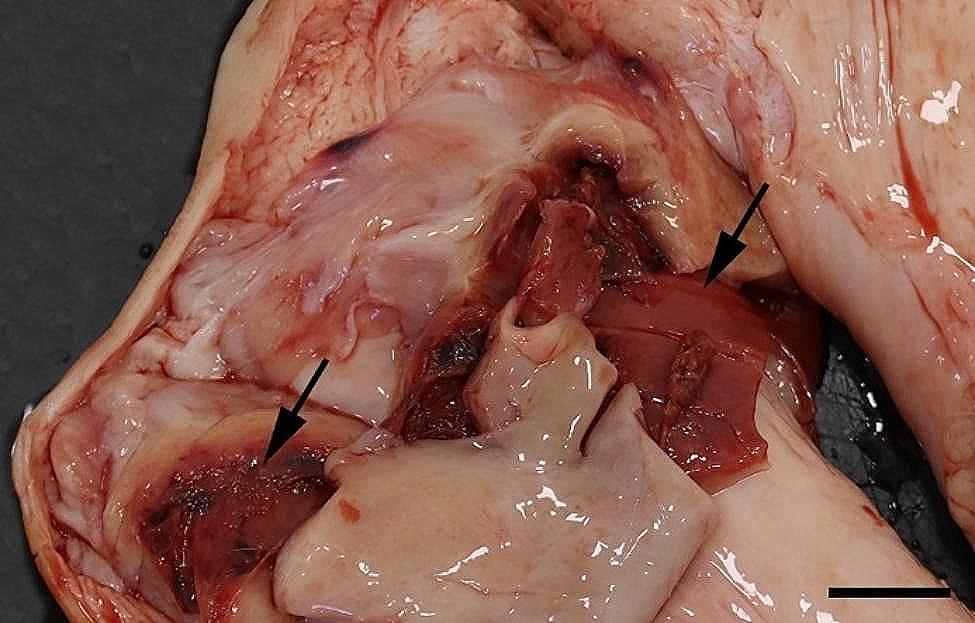
In the hernial cavity of a slaughter pig more hematomas (arrows) are present. Bar = 2 cm

**Fig. 4:**
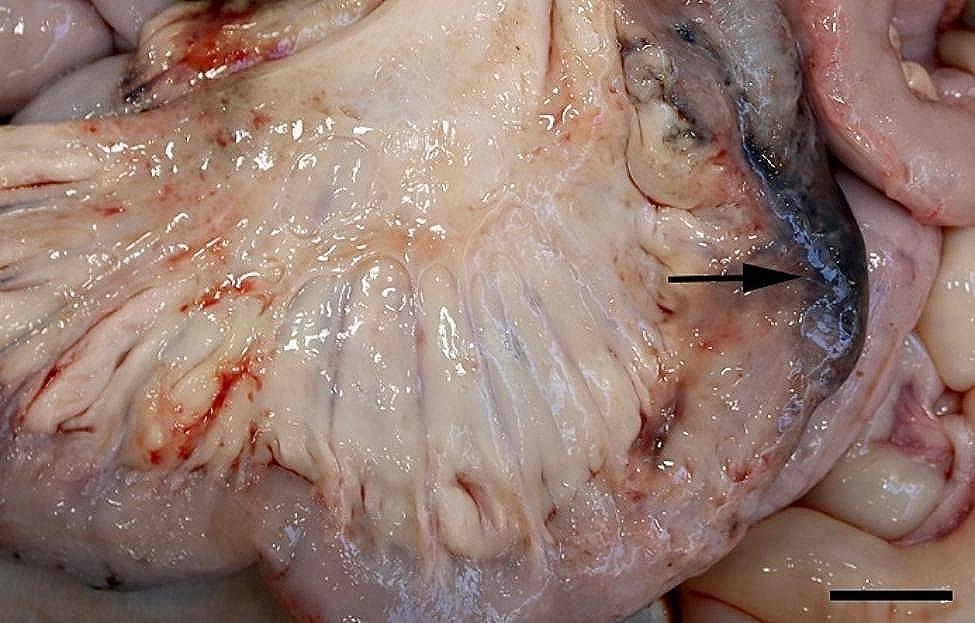
In a slaughter pig with a herniating enterocystoma the serosal lining of the jejunum (arrow) is discolored due to hemosiderosis. Bar = 4 cm

**Fig. 5:**
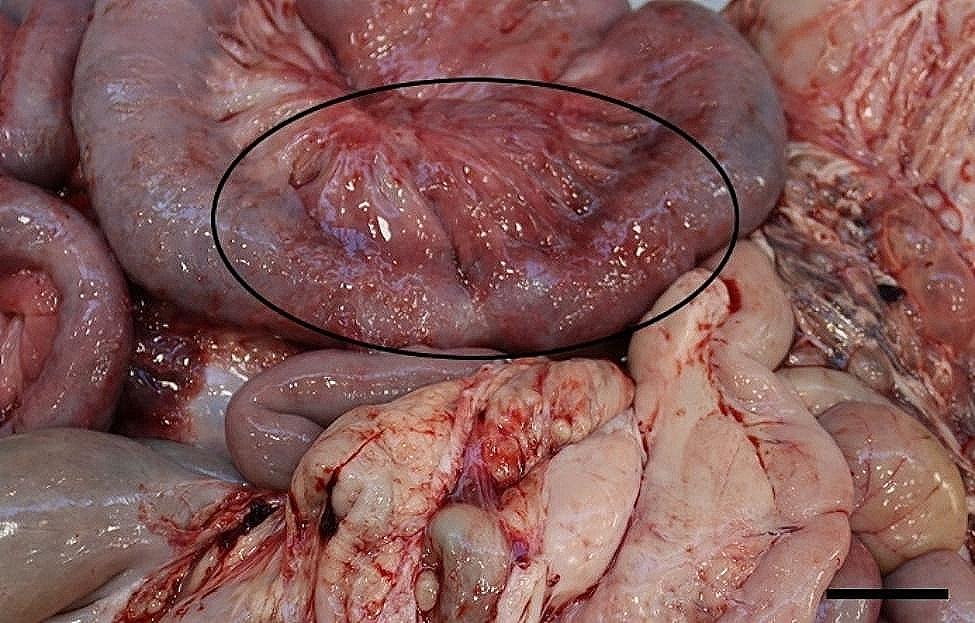
In the mesentery and around a part of jejunum (encircled) of a slaughter pig with an enterocystoma chronic, fibrous peritonitis is present. Bar = 4 cm

**Fig. 6:**
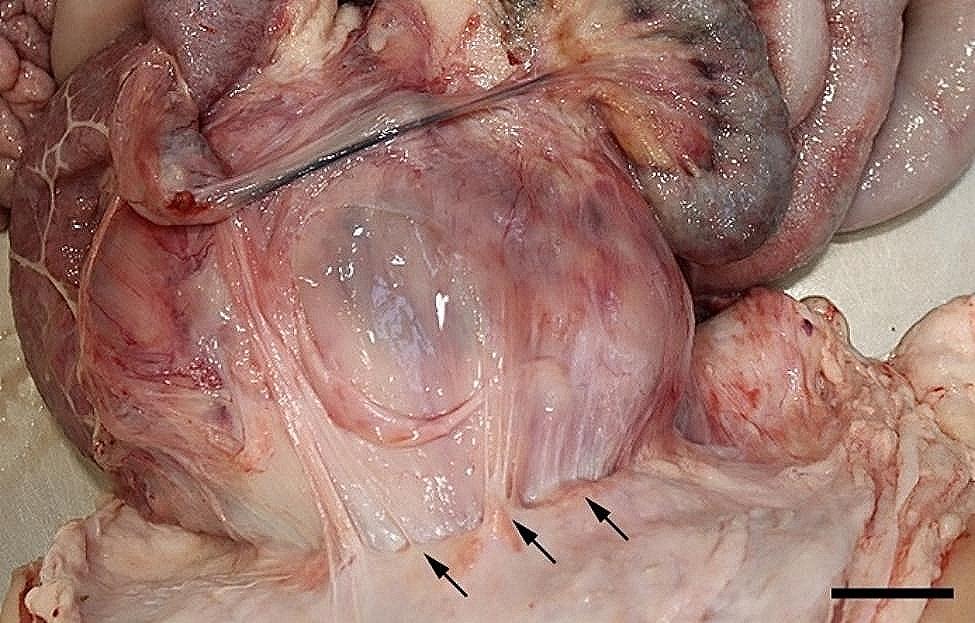
Chronic, fibrous, adhesive peritonitis is present between the serosal lining of the hernial cavity and the jejunum (arrows) of a slaughter pig with a herniating enterocystoma. Bar = 4 cm

**Fig. 7:**
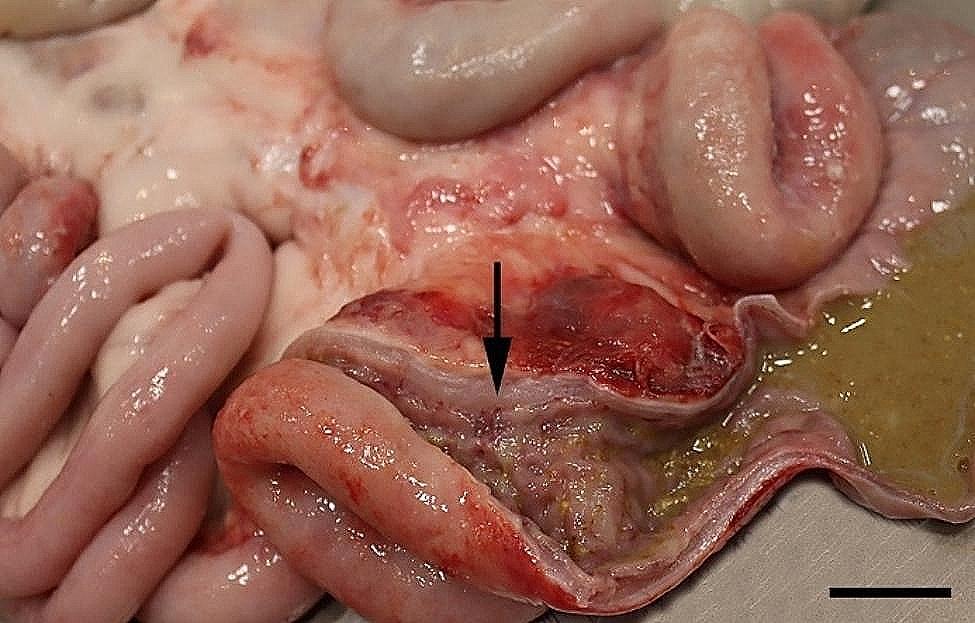
Segmental muscular hypertrophy of jejunum (arrow) is present together with overlaying hemorrhages in the jejunal mesentery of a slaughter pig with an umbilical hernia. Bar = 4 cm

**Fig. 8:**
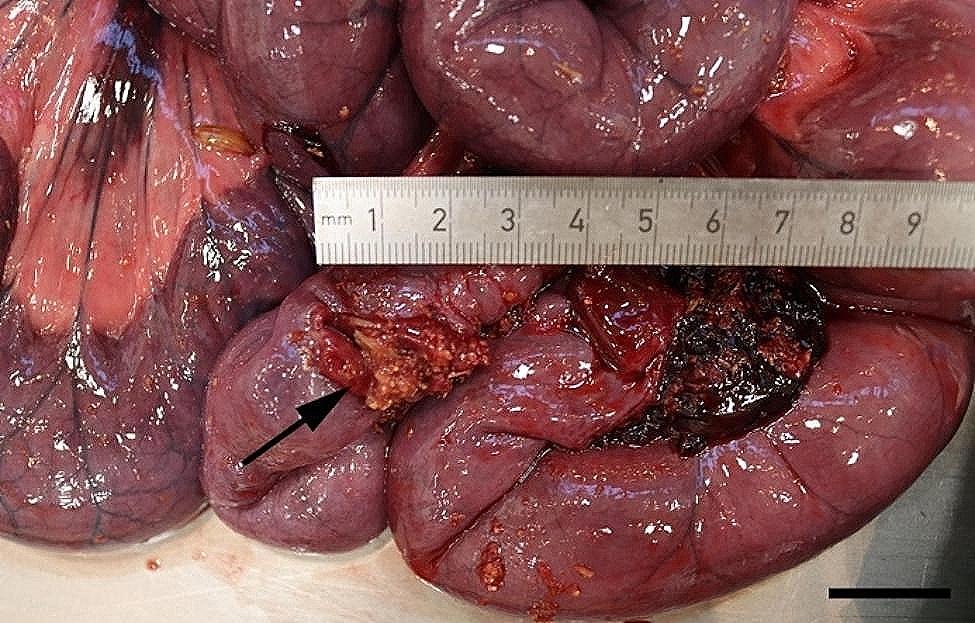
In a slaughter pig with an umbilical haernia acute rupture of jejunum accompanied by hemorrhage and exudation of fibrin (arrow) is present. Bar = 2 cm

**Fig. 9:**
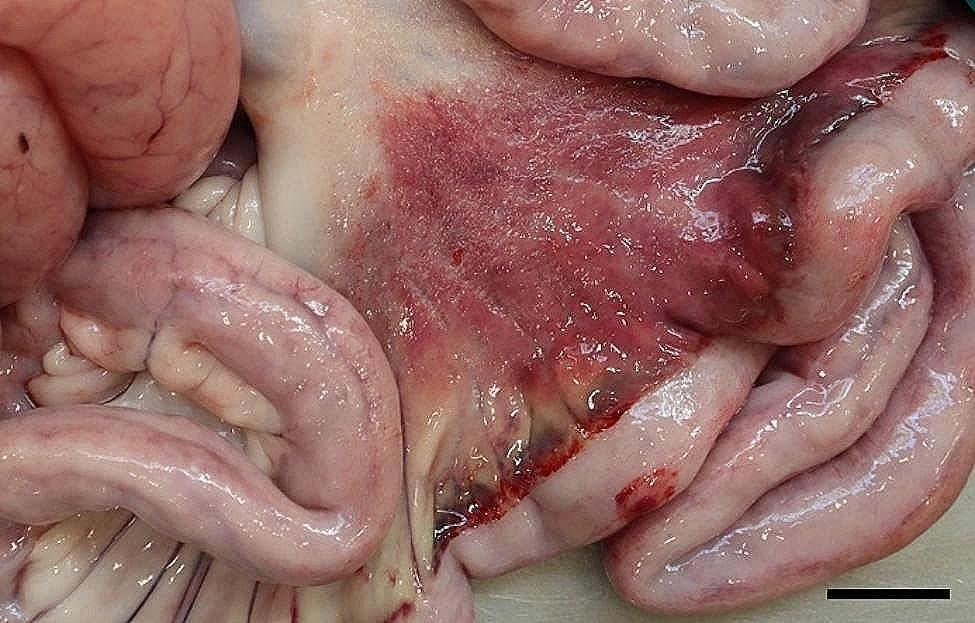
In a slaughter pig with a herniating enterocystoma an area of acute, fibrinous peritonitis is present on the serosal lining of the jejunal mesentery. Bar = 4 cm

All 96 animals fulfilled the clinical criteria for transportation, i.e. they were in a general good condition and the UO were not distended (hard), warm, or associated with pain at palpation. Of the UOs 43 (45%) were elongated and 53 (55%) were spherical. The contents of the UOs were reducible in 58 (60%) of the animals. The wounds on all UOs were dry and less than 4 cm in diameter. At necropsy, 68 (71%) were categorized as umbilical hernias, 13 (13%) as enterocystomas, and 15 (16%) as herniating enterocystomas. Intra-abdominal lesions were observed in 72% (69/96) of all cases (in 65% (44/68) of hernias, 77% (10/13) of enterocystomas, and 100% (15/15) of the herniating enterocystomas). Of the wounds 80% (*n* = 77) were chronic with content of granulation/scar tissue, whereas the remaining 20% (*n* = 19) were acute to subacute. The dimensions of the wounds with respect to the different causes of the outpouchings are stated in Table 1. Hemorrhages were the most frequent finding in all three categories, ranging from a few petechial/dotted bleedings to multifocal/coalescing ecchymosis, which were most often appearing in the peritoneal lining of the outpouchings and in the serosal lining and the mesentery of the jejunum (Figs. 1 and 2). Hematomas were only present within the cavity of the UOs (Fig. 3), and hemosiderosis (Fig. 4) was seen as focal areas of black discoloration of the serosa and were mainly situated on the jejunal segment in relation to other lesions. Chronic peritonitis was also a frequent observation in pigs of all three categories (18% (12/68) in umbilical hernia, 31% (4/13) in enterocystomas, and 73% (11/15) in herniating enterocystomas). Lesions associated with chronic peritonitis were ranging from small foci of peritoneal fibrous thickening to extensive involvement of several intestinal segments with widespread fibrosis, roughening of the serosal surface and fibrous adhesions (41% of chronic peritonitis cases) (Figs. 5 and 6), both between intestinal segments and to the parietal peritoneum. Segmental hypertrophy of the intestinal muscular layers was present in 16 cases. This was solely present in parts of the small intestine (Fig. 7), and it was seen in nearly half (47% (7/15)) of the cases with a herniating enterocystoma, while it was less frequently present in cases of enterocystomas (15% (2/13)) and umbilical hernias (10% (7/68)). Acute lesions, in the forms of acute rupture (intestinal or peritoneal) and/or acute peritonitis (Figs. 8 and 9) were registered in 13 pigs and were only observed in cases with umbilical hernias and herniating enterocystomas. Here it showed a frequency of 15% (10/68) and 20% (3/15), respectively. In a single case of peritoneal rupture of an umbilical hernia, the rupture extended through to the surface of the skin, causing a complete perforation of the UO.

**Table 1:** Intra-abdominal lesions associated with different causes of umbilical outpouchings

Categories and the mean dimensions (± SD) in cm of umbilical outpouchings	Hernia *N* = 68 Mean dimensions: 15.55 × 13.71 (± 2.97 × 2.62)	Enterocystoma *N* = 13 Mean dimensions: 14.38 × 12.92 (± 4.02 × 4.48)	Herniating enterocystoma *N* = 15 Mean dimensions: 17.23 × 14.37 (± 3.52 × 2.61)	Total *N* = 96
**Size of wounds on outpouchings in cm (± SD)**	2.95 × 2.03 (± 1.28 × 0.89)	3.19 × 2.26 (± 1.24 × 1.45)	3.21 × 2.44 (± 1.76 × 1.07)	
**Lesions**				
***Hemorrhage***:				
Peritoneum (outpouching)	30	0	11	41
Peritoneum (intra abdominal)	5	2	7	14
Intestines incl. mesentery	21	2	10	33
Cystic structures	-	6	6	12
***Fibrin***:				
Peritoneum (outpouching)	7	0	1	8
Peritoneum (intra abdominal)	0	0	1	1
Intestines incl. mesentery	3	1	3	7
Cystic structures	-	1	1	2
***Hemosiderin***:				
Intestines incl. mesentery	3	0	2	5
***Hematoma***:				
Peritoneum (outpouching)	4	0	1	5
Peritoneum (intra abdominal)	0	0	0	0
Intestines incl. mesentery	2	0	0	2
Cystic structures	-	1	0	1
***Acute peritonitis***				
Intestines incl. mesentery	5	0	2	7
***Chronic peritonitis***:				
Peritoneum (outpouching)	7	0	5	12
Peritoneum (intraabdominal)	3	3	7	13
intestines incl. mesentery	10	3	10	23
***Adherences***:				
Peritoneum	4	3	4	11
Small intestines	2	2	2	6
Large intestines	0	1	0	1
***Rupture***:				
Intestines incl. mesentery	2	0	0	2
Outpouching peritoneum	4	0	1	5
***Intestinal muscular hypertrophy***:				
Duodenum	1	0	0	1
Jejunum	4	2	7	13
Ileum	2	0	0	2

**Fig. 10:**
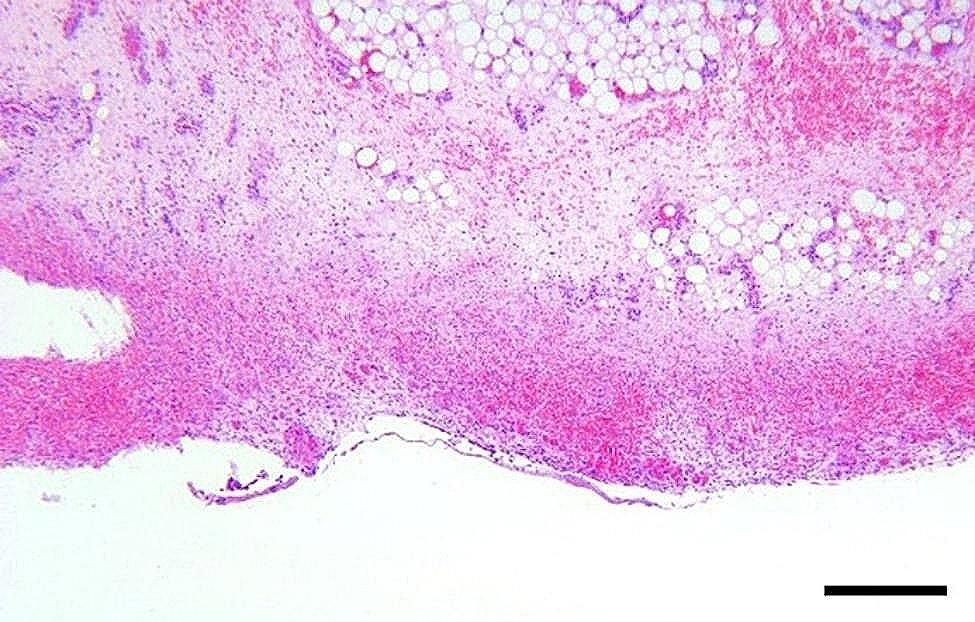
In slaughter pigs with umbilical outpouchings, the presense of acute hemorrhages within the intestinal mesentery was histologically confirmed by the presence of blood intermingled with the mesentery lipocytes. Bar = 400 μm

**Fig. 11:**
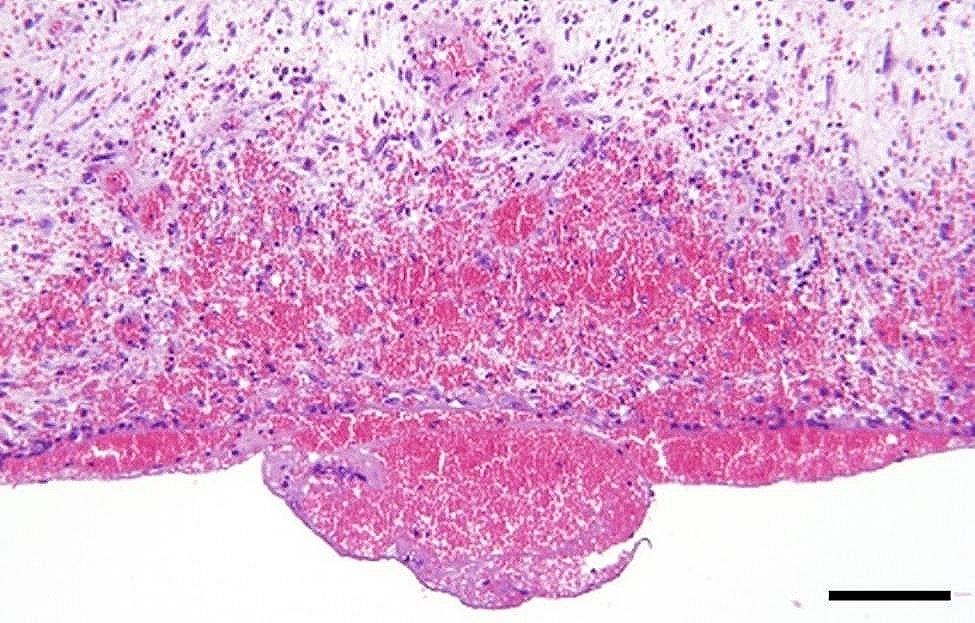
In slaughter pigs with umbilical herniation and herniating enterocystomas the presence of acute, hemorrhagic peritonitis was confirmed histologically by the influx of neutrophils within and on the surface of the mesentery of jejunum. Bar = 200 μm

**Fig. 12:**
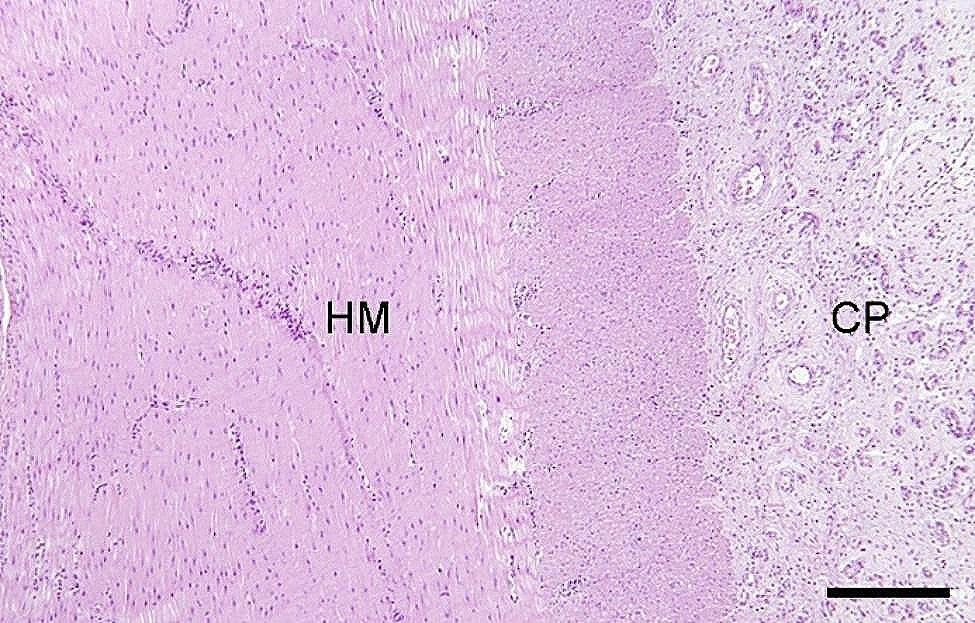
In slaughter pigs with umbilical outpouchings intestinal muscular hypertrophy may be present. In the present porcine case of an enterocystoma, hypertrophy of tunica muscularis externa (HM) is seen together with fibrotic/ granulation tissue (chronic peritonitis (CP) on the serosal lining. Bar = 250 μm

**Fig. 13:**
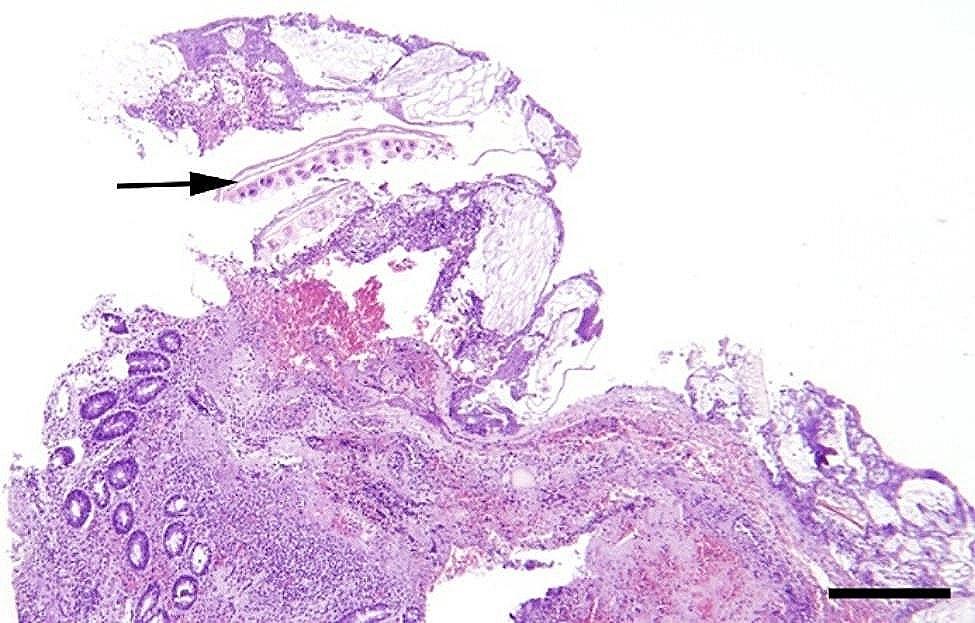
In a slaughter pig with an umbilical hernia, an acute rupure of the jejunum was present. Histologically, within and around the rupture hemorrhage, exudation of fibrin and influx of neutrophils are seen to be accompanied by the presence of intestinal contents on the serosal lining (arrow). Bar = 450 μm

In a majority of the intestinal samples, histopathology revealed hemorrhage in one or multiple layers of the intestinal wall and in the mesentery (Fig. 10). Fibrin exudation to the serosal surface of the intestines was found in cases associated with inflammation. The presence of acute peritonitis was confirmed by the occurrence of hemorrhage, in combination with fibrin exudation and leukocyte infiltration on the intestinal serosa and mesentery (Fig. 11). When chronic peritonitis was present, a thickening of the intestinal serosa and/or mesentery was observed with formation of fibrosis and/or granulation tissue (Fig. 12). In intestinal ruptures, hemorrhage and neutrophilic inflammation was observed at the edges of the perforation extending into the intestinal wall, and intestinal contents were observed on the surface of the intestines (Fig. 13). Intestinal muscular hypertrophy was present in all cases as a segmental thickening of the circular layer of tunica muscularis externa (Fig. 12).

## Discussion

The majority of pigs with UOs showed intra-abdominal lesions (72%), regardless of the appointed category. In a study by Schild et al. (2015), behavioral changes in pigs with UOs at a pick-up facility were observed [9]. These included increased sitting time and decreased latency to lie down, especially in pigs with umbilical hernias. These behavioral changes are indicative of an increased discomfort, hence suggesting pigs with UOs to be less fit for mixing during transportation. The pigs in the present study were all deemed eligible for transportation due to a specific and single time permission from The Animal Experiments Inspectorate of Denmark, in order to evaluate the occurrence of lesions in transported pigs with UOs, which has hitherto not been elucidated. Obviously, further studies are required in order to evaluate the impact of transportation of pigs with the different types of UOs, the size of these and also without the presence of skin wounds on these.

Acute lesions, i.e. acute peritonitis and/or rupture, were found in 13 pigs and observed in two out of the three categories with 15% of the hernias and 20% of the herniating enterocystomas. These acute lesions have a short timeline, and they are compatible with having developed within a few hours before slaughter. This could indicate that some animals may have an increased risk of injury during the process of transport. In this study no acute lesions were found in pigs with enterocystomas. However, due to the low number of pigs with acute lesions more research in needed to investigate whether the presence of a hernial opening is a risk factors for UO pigs to develop acute lesions.

A high number of chronic lesions, e.g. chronic peritonitis and adhesions was found in all categories of UO-animals. The cause of peritonitis may be infectious and non-infectious. When infection is the cause, it may be due to bacterial penetrating through the umbilicus, the wounds on the UOs and/or from lesions of the intestines. The latter, especially when an intestinal perforation/rupture is present. Non-infectious causes of peritonitis are due to excessive stasis leading to extravasation of fibrinogen, which is converted into fibrin and eventually into fibrotic scar tissue. However, in the present study microbiological cultivation attempts were not carried out. Studies of intra-abdominal adhesions in humans post-surgery have shown that patients consider adhesions as a painful affliction [10, 11], which is likely also to apply for pigs. Therefore, movement, strain and physical impacts associated with transportation, therefore, may cause amplification of the pain and discomfort for the animals.

The pigs in the present study all had wounds on the UOs. However, it is likely that animals without wounds on the UOs would present with similar types and frequencies of lesions, following the transportation-process including the physical stressors from staying in a pick-up facility, loading and unloading, and the drive at the abattoir. However, to extrapolate the findings in this study to UO pigs in general more knowledge is needed regarding the prevalence and severity of intra-abdominal lesions in UO pigs without wounds.

The transportation time for the included animals did not exceed 2 h, which also complied with the Danish transport regulations stating that pigs with UOs that are approved for transport, must be directly transported to an abattoir in close proximity to the farm of origin. However, the regulation does not specify a maximum transportation time. Longer transportation time than in the present study, is therefore also likely to increase the risk of developing or exacerbating UO-associated lesions in pigs.

## Conclusions

Based on the present study, the prevalence of intra-abdominal lesions in pigs transported with a wounded UO was found to be high independent of the underlying condition, and it is clear that these pigs possess a vulnerable group of animals, especially during physical stressful situations like the process of transportation. The results provide valuable knowledge, that can be used when examining and evaluating UO pigs before transport, so especially acute lesions associated with the process of transportation can be reduced. In future studies the impact of transportation of pigs with different sizes of UOs, causes of UOs, and UOs without wounds should be evaluated, in order to enhance the welfare of these vulnerable animals.

## Methods

This study was designed as a cross-sectional study of finisher pigs transported to slaughter with wounds on the UOs. The data were collected from three Danish conventional swine herds from January to June 2023.

A total of 96 Danish crossbreds (Landrace/Yorkshire x Duroc) were included. Before pigs were included, a thorough clinical examination by a veterinarian was carried out the day before transportation and slaughter. The assessment of fitness for transport under special circumstances included that the animals should be in general good condition and the UO should not be distended (hard), warm, or associated with pain at palpation. The shape of the UOs were recorded together with the reducibility of the contents. The wound on the UO had to be dry and less than 4 cm in diameter. Transportation time to the abattoir had a maximum of 2 h.

On the transportation lorry, the pigs with UOs were placed in groups of 5 to 7, which were separated from other pigs, and they had extra room and bedding in accordance with guidelines provided by The Danish Veterinary Health Council, 2008 [12]. At the abattoir, standard slaughter procedures were carried out, and the UOs together with the gastrointestinal tract were transported to pathologic examination, which was performed within 6 h after slaughter.

When arriving at the University of Copenhagen for pathologic examination, the UOs together with the gastrointestinal tract, were registered by the ear tag number and given an individual journal number. In order to ensure documentation of all lesions, the UOs were standardly photographed, with an overview of the dorsal and ventral surfaces. In addition, an overview photo was taken of the intestines, and the content of the UOs.

The dimensions (length and width) of the UOs were recorded. The UOs were categorized as either (1) an umbilical hernia, a sac formed by a pouch of parietal peritoneum protruding outside the abdominal cavity, (2) an enterocystoma, a cystic congenital malformations due to a non-regression of the yolk-sac, or (3) a herniating enterocystoma, i.e. a combination of the two, comprising a multi-cystic structure and a hernial ring [13, 14] A thorough pathological examination was carried out, and the intra-abdominal lesions were classified as either bleedings (hemorrhage (acute widespread bleeding), hematomas (acute or chronic localized accumulation of blood), and hemosiderosis (accumulation of hemosiderin)), inflammation (acute and chronic peritonitis), adherences (connective tissue formation connecting separate organs), ruptures, and/or hypertrophy of the intestinal musculature (Table 1). During necropsy, tissue samples were collected from the different lesions for histological examination. Tissue samples were fixed in 10% neutral buffered formalin, and processed routinely for histopathology and stained by hematoxylin and eosin.

## Data Availability

No datasets were generated or analysed during the current study.
